# Minimally Invasive Hemodynamic Assessment during Obstetric Hysterectomy for Invasive Placentation with Epidural Anesthesia

**DOI:** 10.1155/2020/1968354

**Published:** 2020-10-28

**Authors:** S. Alvarado-Ramos, V. J. Lara-Díaz, M. R. López-Gutiérrez, M. E. Torcida-González, J. F. Campos-Rodríguez

**Affiliations:** ^1^Unidad Médica de Alta Especialidad Hospital de Ginecología y Obstetricia, No. 23. Instituto Mexicano Del Seguro Social, Monterrey, Nuevo León, Mexico; ^2^Tecnológico de Monterrey, Escuela de Medicina y Ciencias de La Salud, ITESM, Monterrey, Nuevo León, Mexico; ^3^Anesthesiology Resident, Monterrey, Nuevo León, Mexico

## Abstract

**Background:**

The present study aimed to describe the evolution of hemodynamic parameters over time of patients with invasive placentation during their third trimester who were delivered via cesarean section and subsequently underwent obstetric hysterectomy under epidural anesthesia.

**Methods:**

A prospective, descriptive, longitudinal, 11-month cohort study of 43 patients aged between 18 and 37 years who presented with invasive placentation. Minimal invasive monitoring was placed before the administration of epidural anesthesia for hemodynamic parameter tracking during the cesarean section. After delivery, the patients underwent an obstetric hysterectomy. Blood loss, hemodynamic parameters, and coagulation were managed via goal-directed therapy. Parameters were compared via repeated measures ANOVA and effect size estimation (Cohen's d).

**Results:**

The mean age of the patients was 29.2 ± 3.4 years and was moderately overweight. They had minor cardiac index variance (*P*=NS, no significance), vascular systemic resistance index (NS), heart rate (*P*=NS), and median arterial pressure (*P*=NS). Differences were observed in the stroke volume index (*P*=0.015) due to moderately higher values (*d* = 0.3, *P*=0.016) in the middle of the surgery. Patients had lower cardiac index (*d* = −0.36, NS) and cardiac workload requirements (*d* = −0.29, *P*=0.034) toward the completion of surgery.

**Conclusion:**

Patients who are in their third trimester and who subsequently underwent obstetric hysterectomy under epidural anesthesia had modest surgical hemodynamic variance and reduced cardiac workload requirements toward the end of the surgery.

## 1. Introduction

During the third trimester of pregnancy, hemodynamic adaptations must be developed to achieve increased cardiac workload, thereby providing the metabolic demands of a growing fetus. At this time, cardiac output increases by 20%–30% than the prepregnancy values, leading to a hyperdynamic state [1, 2]. In pregnancies with invasive placentation, the need for total hysterectomy increases the risk of massive obstetric hemorrhage, with a potential blood loss of approximately 3000–4000 mL, which results in further hemodynamic adjustments, increases metabolic stress, and causes dilutional or consumptive coagulopathy [3, 4].

The optimal anesthetic approach for these surgical events remains to be fully elucidated since existing reports are of observational design rather than experimental. Moreover, performing clinical trials in this patient population is ethically questionable. Traditionally, the use of general anesthesia is preferred instead of regional anesthesia since it maintains steady hemodynamic parameters, rather than the sympathectomy-like status, as observed after the administration of neuraxial anesthesia and, hereby, additional hemodynamic adjustments or vasopressor management [5]. Nguyen et al. found no differences in the results of retrospective cohort studies about blood loss, the requirement for transfusion, nor variances in neonatal outcome regardless of the anesthetic technique [6]. Other case reports, case series, and cohort studies may indicate that catheter-based regional anesthesia is a possible alternative. Evidence regarding hemodynamic maternal adaptations to neuraxial anesthesia is focused on either vaginal delivery or cesarean section with limited information about critical conditions.

Most information about pregnancy cardiac function derives from the reports produced in the 1970s and 1980s; those studies used a pulmonary artery catheter, allowing high measurement reliability. Since the early 1990s, publications about minimally invasive or noninvasive technologies emerged, such as impedance, arterial curve flow interpretation, and echocardiography. The complexity reduction for cardiac function evaluation is of exceptional assistance for immediate clinical decision making. Nevertheless, the technologies are still in development and had several limitations regarding the patient body mass index (BMI > 30 kg/m^2^) [7], reduced accuracies during high or inadequate cardiac output [8, 9], and impaired concordance in patients with high endothelial permeability and vasodilation like the septic shock status [10].

Our study gathered data about the vascular flow parameters approximations and cardiac function during the obstetric hysterectomy, as well as the adjustments presented to the use of neuraxial anesthesia.

### 1.1. Research Design

This study is a prospective, descriptive, longitudinal, 11-month cohort study of 43 patients with invasive placentation who underwent an obstetric hysterectomy.

## 2. Objectives

This study primarily aimed to describe the hemodynamic approximations of patients who presented with invasive placentation during their third trimester. The patients were delivered via cesarean section and subsequently underwent obstetric hysterectomy with epidural anesthesia. As a secondary objective, we evaluated the variations in the hemodynamic approximations during the surgery.

## 3. Methods

### 3.1. Patient Selection

The National Bioethics Committee CONBIOETICA-19-CEl-011-20161017 and Monterrey Institute of Technology and Higher Education Research Committee 13Cl19039138 reviewed and approved the research protocol. The manuscript is a synopsis of the Thesis “*Evaluación Hemodinámica Mínimamente Invasiva Durante Histerectomía Obstétrica con Bloqueo Epidural por Invasión Placentaria*” [11].

Our center had 15 445 live births in 2018. In that course of time, 670 (4.33%) patients developed obstetric hemorrhage, and 70 (0.45%) developed massive hemorrhage, where invasive placentation characterized the majority of those events. The center gathers patients with abnormal placentation from six northern Mexico states. Patients with invasive placentation or with expected blood loss above 50% of the predicted blood volume during their cesarean section are followed by the high-risk pregnancy care team. It includes physicians from Gynecology, Anesthesia, Neonatology, and Intensive Care specialties, who offers advice and consultation to both patients and caregivers. The anesthesia team practices a regional (epidural) approach given the related gastric aspiration risks during general anesthesia induction and diminishes the pharmacological exposure of the fetus. All patients, either in this report or in regular clinical practice, are managed with goal-directed therapy, minimally invasive hemodynamic follow-up, and point of care coagulation assessment to ensure patient safety, regardless of the anesthesia technique provided. The present manuscript reports the observations of the hemodynamic approximations on those prospective mothers who selected regional (epidural) anesthesia as their first option for their surgery.

The protocol included elective patients aged between 18 and 39 years who were diagnosed with invasive placentation (placenta accreta, increta, or percreta) and subsequently underwent a hysterectomy at a gestational age of 34–36 weeks, from April 2018 to February 2019. Exclusion criteria were infectious diseases, nonreassuring fetal status, endocrine dysfunction, pulmonary hypertension, congenital or acquired heart diseases, coagulopathy, preeclampsia or HELLP syndrome (Hemolysis with a microangiopathic blood smear, Elevated Liver enzymes, and a Low Platelet count), emergency c-section, and those who refused a regional anesthesia technique. Participants with incomplete records were not included.

Patients with invasive placentation were diagnosed via ultrasonography and were hospitalized 10 and 20 days prior to their scheduled surgery. They were assessed by an anesthesiologist the day before surgery for consultation, updating blood test results, and actualization of medical records. The anesthesiologist also reviewed the operation phases, anesthesia technique alternatives, and in some instances, the conversion to general anesthesia in those patients who selected a regional approach. The anesthesia and surgery were explained to the patients and all participants signed written consent.

Before surgery, patients received antiemetic prophylaxis (Ranitidine 50 mg/Metoclopramide 10 mg IV) and vitamin K (10 mg IV) and verified the availability of blood products. The medical team (three surgeons, two anesthesiologists, and three registered nurses) briefed the surgical plan.

### 3.2. Monitor Setup

Before the epidural technique, the anesthesiologist set up the minimally invasive monitor (EV1000 G 1.9, Edwards Lifesciences, Irvine, USA) with the patient's height, weight, age, and biological sex. The patient profile, together with the interpretation of the arterial curve features, allows the EV1000 software to produce hemodynamic approximations. The radial arterial line with a 22-G catheter was placed after a negative Allen's test. At the same time, the second anesthesiologist placed an ultrasound-guided triple lumen central venous 7-Fr catheter (Multi-lumen CVC, Arrow International, Reading, PA, USA) in the internal jugular vein with a 6–13 Mhz linear transducer. The arterial line (FloTrac, Edwards Lifesciences, Irvine, USA) and the distal hub of the central catheter were connected then to the minimally invasive monitor for cardiac index (CI, L/min/m^2^), systemic vascular resistance index (SVRI, dynes·sec/cm^5^/m^2^), stroke volume index (SVI, mL/beat/m^2^), central venous pressure (CVP, mmHg), mean arterial pressure (MAP, mmHg), stroke work index (SWI, gr × min/m^2^ [SVI ×MAP ×0.0144]), and cardiac work index (CWI, kg × m/m^2^ [CI ×MAP ×0.0144]) tracking. Before the interpretation of these values, calibration of both arterial and central line to atmospheric pressure took place with the transducers at the thoracic level.

Next, the anesthesiologist installed a lumbar (L2–L3) epidural (Touhy 18) catheter (20 G), and after a negative test dose (lidocaine 60 mg/epinephrine 15 mcg), 10 mL of 2% lidocaine and 10 mL 0.75% ropivacaine were administered to achieve a T3–T2 sensory block level (assessed using cold sensation). The surgical team does not place ureteral catheters; instead, a urogynecologist supports the staff. This measure is to shorten the fetus's delivery; thus, the C-sections started 20 min after the administration of epidural anesthesia. Because of infrastructure limitations in the OR, internal iliac artery catheterization by interventional radiology is not carried out. Alternatively, the surgeons implement the vascular interventions on-site.

### 3.3. Cesarean Section and Hysterectomy

The fetus was delivered via corporeal hysterotomy. The surgical team practiced uterine artery ligation to reduce the organ blood flow. They performed also hypogastric artery ligation if placenta percreta was present. Once the fetus was delivered, the anesthesia care team administered carbetocin 50 *µ*g IV in 90 s to increase the uterine tone and minimize blood flow and the specimen volume during this phase.

Hemodynamic variations as a consequence of the effects of the epidural anesthesia, carbetocin, or bleeding were treated immediately with ephedrine (5 mg IV) or balanced crystalloids (300 mL bolus of Ringer Lactate) when the mean arterial pressure (MAP) decreased below <65 mmHg or SVRI under <1200 Dyn. Once ≥2000 mL of Ringer Lactate was infused, the fluid therapy changed to Ringer Lactate + 4% albumin [12–14].

The coagulation goals were directed via viscoelastic thromboelastography (TEG 5000 Thrombelastograph Hemostasis Analyzer System, 4.02.101, Haemonetics Corp., Braintree, MA, United States) with fresh frozen plasma (R (reaction time) >8 min, 10–15 mL/kg [15]) or cryoprecipitate (MA (maximum amplitude) <60 mm, 2 mL/kg [15]). Goal hemoglobin values were at >80 Hb g/L (Table 1). At the end of the surgery, 2 mg of epidural morphine was administered for postoperative analgesia. Blood samples for hematic cytometry, coagulation tests, and blood chemistry were collected.

### 3.4. Data Management and Statistical Analysis

The research team recommended at least a sample size of 43 patients to attain precision (Z*α*/*β* = 1.96/1.036) in the planned analysis. The estimation focused on achieving a proper resolution with cardiac index modifications as low as 1 L/m^2^ (*d*), in a known variance of 1.5 L/m^2^ (*σ*), estimated from previously baseline readings of 22 patients (not included in this report). The patient's clinical information was gathered from the medical archives. Hemodynamic parameters (21 267 row entries, 118 h) were downloaded from the EV1000 monitor to a Universal Serial Bus memory in CSV (comma separated values, *∗.csv*) format with single measurements every 20 s; later, these records were cleansed by removing entries without stored values or artifacts. Finally, time adjustment was conducted then for all series. The gathered data readings were used at time 0, the phase before the epidural block. Baseline recordings were generated from this stage since no pharmacological effects or surgical interventions are present.

**Figure 1:**
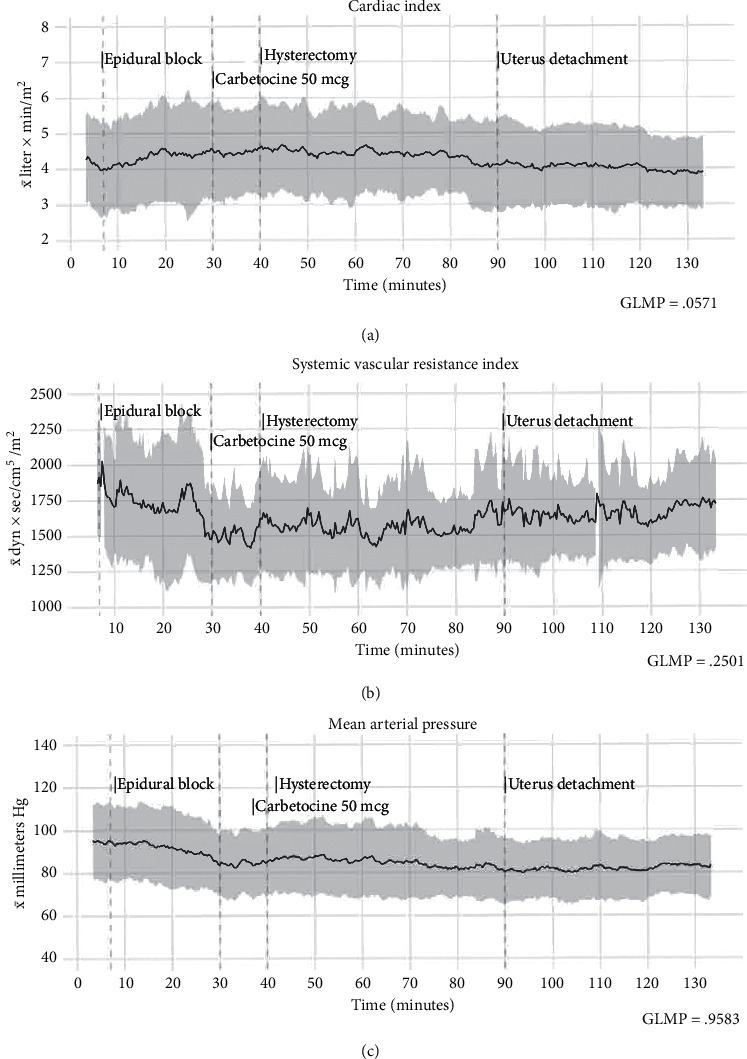
Hemodynamic trends (x̄, bold black line; *σ*, gray zone). Cardiac index (a) initially showed a mild hyperdynamic pattern, followed by an increase due to the epidural block, and it remained relatively stable during the surgery, with inferior values at the end when compared with the first ones. The systemic vascular resistance index (b) decreased after the epidural block. However, these fluctuations were maintained with minimal variance despite the bleeding. Mean arterial pressure (c) showed less variation and did not exhibit the changes recorded in the vascular resistance index.

**Figure 2:**
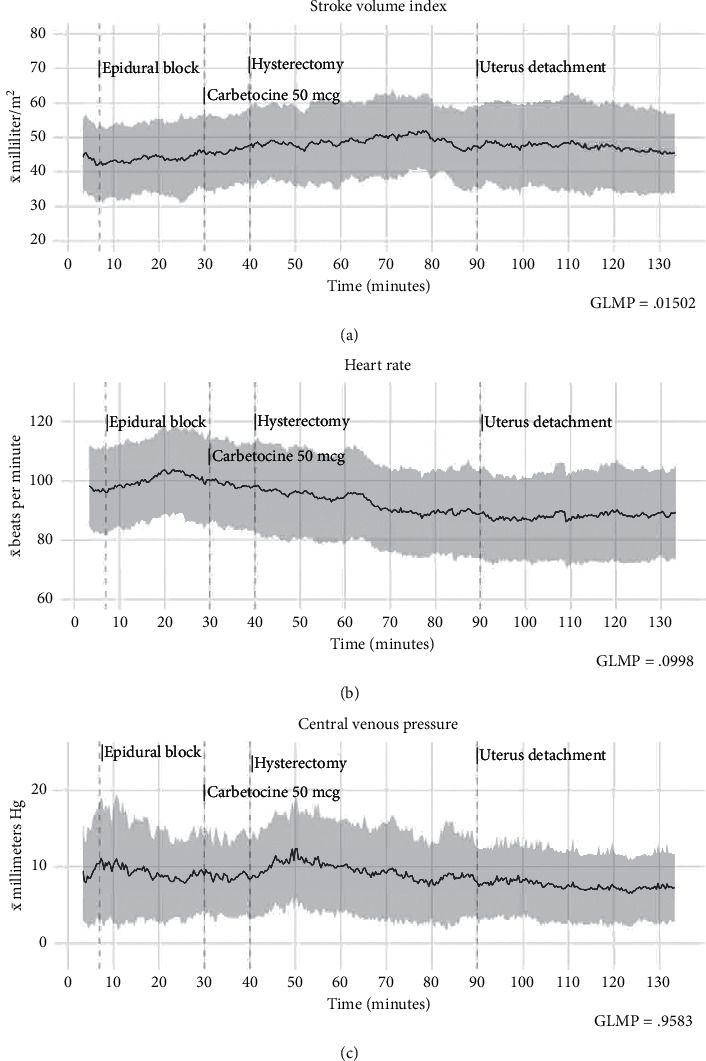
Hemodynamic trends (x̄, bold black line; *σ*, gray zone). (a) Increased variance (*P*=0.015) in the stroke volume index occurred within the measurements; the pattern could be explained by an increase in the variability between the individuals; however, these variances were more noticeable when the effect size (Cohen's d) was measured after 60 and 75 min. The heart rate (b) was expected to change due to cardiac and vascular adaptations caused by the epidural block neurovegetative effects and featured mild variance throughout the surgery. Central venous pressure (c) showed minor variations during the procedure. Initial increments correspond to the patients' lateral decubitus position for the epidural block.

Data organization and computation was carried out using the *R* 3.5.1 environment [16]. The evaluations included descriptive summaries (mean and standard deviation) at time stages (0, 5, 15, 30, 45, 60, 75, 90, 105, 120, and 135 min) for the tabular report and for the respective time entry (3 per minute) for plotted data (Figures 1 and 2. x̄, bold black line; *σ*, gray zone). The selected time stages consisted in postcalibration and baseline parameters (0–5 min); epidural block and mother's vascular adaptations to the epidural anesthesia (5–15 min); c-section, delivery, and uterotonic effects (15–30 min); and the hysterectomy stage (45–120 min). Comparative analysis of the time series was performed with general linear modeling for repeated measures (repeated measures ANOVA). The “Pillai–Bartlett” trace variance analysis was used to counterbalance the F statistic when multiple observations have nonhomogeneous covariance within the sequential measurements. The hemodynamic parameters were analyzed then at 11 times stages (0, 5, 15, 30, 45, 60, 75, 90, 105, 120, and 135 min). *P* values < 0.05 were considered significant.

Post hoc analyses were produced with a one-tailed paired *t*-test by a hypothesis assumption that discrepancy would be observed per time stage with respect to the initial recordings. Therefore, the calculations would characterize what period (clinical conditions surrounding it) may confer high variance in the sample from the perspective of the initial phase (0 min). Post hoc *P* values < 0.025 were considered significant.

**Figure 3:**
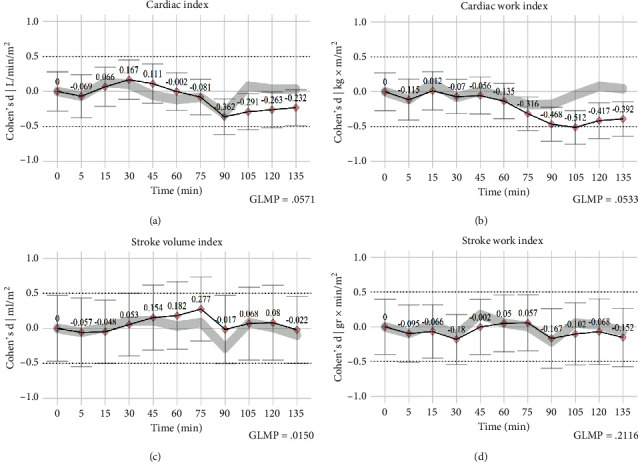
Effect size (Cohen's d) assessment. The bold black line represents the changes (Cohen's d) concerning the initial measurements; the differences between the time intervals are described in gray color. Cardiac index (a) undergoes small initial variations, secondary to vasodilation after epidural block and low variance afterward. At the end of the procedure, a moderate decrease in cardiac output was observed, which corresponds to the removal of the uterus from the systemic circulation. The cardiac work index (b) displayed more substantial changes at the end of the surgery. These fluctuations were followed by a mid-procedure stroke volume index adjustment (c). Despite these hemodynamic modifications, the stroke work index had (d) limited variation. Thus, we hypothesized that the noncardiac parts of the vascular system experience most of the adaptations, and the cardiac work investment is relatively the same for every stroke.

Furthermore, Cohen's d (*d*) value was estimated, with sample size adjustment, to determine the change factor (±0.2 small; ±0.5 medium; ±0.8 Large) between the time intervals. Those calculations occurred (Cohen's d) in two forms. First, we wanted to improve the *P* value information from the one-tailed *t*-test and gain a further understanding of the detected modifications and the baseline readings. This can be found referenced along with the *P* values in the text, also in the manuscript's Figure 3, and in the supplementary material content (Figure S2) plotted as a red diamond dot and the bold black line linking the items. Second, we added another set of calculations (*d*) directed to evaluate the change taking place between the successive period with the immediately previous one and so examine the progressive change factor as the surgery develops; these calculations are plotted in Figure 3 and supplementary content (Figure S2) as a gray line.

## 4. Results

**Figure 4:**
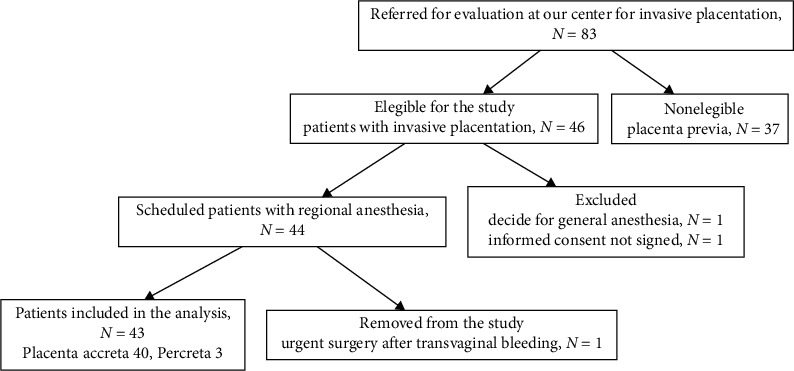
Study profile.

The study evaluated the records of 43 patients with invasive placentation (Placenta Accreta 40, Percreta 3) from 83 possible patients referred to our center for invasive placentation. For more details on the patient selection, observe Figure 4. Patients had a mean age of 29.2 ± 3.4 years, three (1–6) pregnancies, had at least one previous cesarean section, and were moderately overweight (body mass index: 29.8 ± 2.8 kg/m^2^). Demographic summaries are presented in Table 2. They were scheduled between 34 and 36 weeks of gestational age; blood test parameters were within the normal range. All patients had at least three hours of beat-to-beat hemodynamic entries, and the final database had 313 503 measurements.

Overall, after assessing the hemodynamic parameters, we observed no differences in cardiac index (*P*=NS, no significance), vascular systemic resistance index (*P*=NS), heart rate (*P*=NS), and median arterial pressure (*P*=NS) at the sequential trend analysis. Differences were only observed in the stroke volume index (F {6.713}, *P*=0.015) when comparisons were made among the time intervals themselves. Descriptive summaries and the statistics of the general linear modeling for repeated measures are present in Table 3, and the post hoc analyses are in the supplementary material (Table S1). The manuscript describes particular observations in the format *Size of effect* *+* *post hocPvalue* (*d* = *X*, *P*=*X*) for the written report.

### 4.1. Epidural Anesthesia and Delivery

The initial measurements of the patients were characterized by a mild hyperdynamic pattern (CI: 4.5 ± 1.3 L/min/m^2^) as well as a relatively normal vascular systemic resistance. This behavior had minor modifications after 5 min (*d* = −0.07, *P*=NS) and even after the administration of epidural anesthesia (5–15 min), with cardiac index average increments to 4.6 ± 1.6 L/min/m^2^ (*d* = −0.07, *P*=NS) and lower systemic vascular resistance index (SVRI, *d* = −0.12, *P*=NS) as shown in Figure 1. An elevated heart rate constituted the principal vascular adjustment, and it presented a moderate elevation (*d* = 0.266, *P*=0.009) posterior to the epidural anesthesia administration, which raised the cardiac output to compensate for the vascular changes mentioned before. All the while, the cardiac work index (*d* = −0.01, *P*=NS) and stroke work index (*d* = −0.066, *P*=NS) remained almost the same. Only four patients required vascular support with ephedrine 5 mg IV at this point.

At the 30 min interval, we observed a higher than expected cardiac index (*d* = 0.167, *P*=NS). The phenomenon may be explained by the heart rate increase in response (*d* = 0.22, *P*=0.022) to a moderate vascular resistance reduction (*d* = −0.33, *P*=0.043) predominantly produced by the administration of carbetocin 50 *µ*g IV. The vasodilatory effects were observed 50–80 sec after the bolus. At most, eight patients required ephedrine 5 mg IV Bolus after measuring bellow SVRI 1200 Dyn as a consequence of the uterotonic, and the other ones had acceptable cardiovascular adaptations with RL boluses only. The changes observed during this phase also included the mother's emotional response, characterized by a rise in heart rate. Amidst these events, no changes in the cardiac work index (CWI, *d* = −0.07, *P*=NS) or left ventricular stroke work index (LVSWI, *d* = −0.2, *P*=NS) were observed.

### 4.2. Hysterectomy

Patients underwent hysterectomy between 45 and 90 min. Throughout the present timeframe, blood loss, hemodynamic instability, and coagulopathy were the primary concerns; still, we observed almost no changes between the CI, SVRI, or HR. All patients surpassed the RL fluid threshold and alternated the fluid therapy to RL + albumin 4%. Patients (11) who experienced an accelerated decline in MAP or SVRI, most often secondary to blood loss, were given ephedrine 5 mg IV boluses, followed by RL + albumin 4% 300 mL boluses till hemodynamic improvement. Half the patients in the sample required fresh frozen plasma (3–5 units, *R* > 8 min). Furthermore, all episodes receive Cryoprecipitate (2 ml/kg) as a result of early identification of (MA < 60 mm) TEG amplitude alterations around the loss of 50% of the patient's estimated blood volume. The fluid management, as mentioned earlier, is believed to improve the viscosity and colloid osmotic pressure quality of the intravascular compartment. The “*low variance*” observed could be explained mostly via the active decision making managing the fluid and transfusion therapy working together with the surgical expertise as the operation advanced. Patients had low packed red blood cells' requirements (3–4 units) with only one patient requiring 5 RPBC units.

The patients showed a moderately higher SVI volume (*d* = 0.3, *P*=0.016) at the 75 min interval, without other hemodynamic adaptations or interrelated cardiac changes. After the 90 min timespan, a nonsignificant drop to standard cardiac index ranges at 3.9 ± 1.1 L/min/m^2^ (*d* = −0.36, *P*=NS) was noticed (Figure 1). Amidst this timeframe (90–120 min), a complete interruption between the uterine and maternal blood circulation takes place, with the consequent loss of the uterine arteriovenous shunt. Lower demand in cardiac output resulted in a decline in the cardiac work index (105 min, d = −0.29, *P*=0.034) and stroke index action (*d* = −0.51, *P*=0.044), thereby preserving circulatory function at almost the same cardiac effort, with less workload for the heart. The cardiac work behavior described before could be observed with better objectivity in Figure 3.

The anesthesia team followed any hemodynamic change caused by bleeding or surgical stress and gave active management to reach the flow goals documented in the EV1000 monitor. Thromboelastography guided the administration of blood products to sustain a satisfactory coagulation activity, despite significant blood volume loss, with minor differences between the initial and final values of the international normalized ratio (*d* = −0.19, *P*=NS) and activated partial thromboplastin time (*d* = 0.42, *P*=NS) and Δ 1 second in prothrombin time (14.4 s, ±1.2, d = 0.98, *P*=0.017) from perioperative values. More information about coagulation blood tests, hematic cytometry, and blood chemistry is displayed in Table S2 in the supplementary material.

Patients were transferred to the intensive care unit without transfusion-related lung injury (TRALI), transfusion-associated circulatory overload (TACO), or vasopressor support. Also, no patient required conversion to general anesthesia, and no TEG sample developed fibrinolysis. Patients had 48 hours in the intensive care unit and relocated to standard hospital beds later. The discharge from the hospital was after 72 hours of observation. Three patients required abdominal packing owing to friable pelvic endometriosis implants with persistent minor bleeding despite no evidence of coagulopathy by TEG or blood testing. The pelvic gauzes were removed then in the next 24 hours with no incidents.

## 5. Discussion

The maternal hemodynamic profile is a result of the adaptation of the cardiovascular system to the metabolic demands levied by the pregnant uterus [17]. This adaptation includes a set of modifications: an increase in the cardiac index, a reduction in vascular resistance due to increased endothelial production of nitrous oxide, a higher level of prostaglandins (prostacyclins), and an adjustment of the renal and mesangial vasculature for the new vascular fluid load [18, 19].

Similar to other reports of high-risk obstetric patients who underwent surgical procedures during their third trimester of pregnancy, we found hyperdynamic patients with initial values comparable with those described by Dyer et al. [20] and McDonald et al. [21]. The authors describe the preservation of this hyperdynamic pattern throughout the procedure, with a slight increase after the neuraxial block. In these reports and most studies in the field, patients undergo spinal anesthesia; in our paper, epidural anesthesia was preferred.

Our patients adapted to the systemic vascular resistance reduction induced by the regional anesthesia with a higher cardiac output. Others have observed cardiac output increments after neuraxial anesthesia, as shown in the study of Mon et al. [22] and Rosseland et al. [23]. They evaluated patients during the initial stages of surgery and then described the cardiac output variations and vasopressor demands during the delivery. We did observe a rise in the cardiac output after C-section delivery, although not statistically significant, and gradually attenuated to preoperative values by the end of our measurements (135 min). Nonetheless, at the end of the surgery, our patients no longer had a uterine shunt that requires blood flow demand. This condition might explain the gradual decrease (as separation of uterine blood flow occurred) in cardiac index and cardiac work in our study.

Most reports analyze no more than 30 min of hemodynamic parameters after birth, which may explain why the cardiac index adjustments after 2 hours are often unnoticed. In recent years, Kuhn et al. [24] have shown that cardiac output and stroke volume, although with hyperdynamic features during labor, had a slight rise after vaginal delivery with epidural analgesia and progressively returned to prelabor values. In some instances, the return to basal figures (hyperdynamic pattern) was observed as early as 15 min after delivery. Rosseland et al. studied the cardiac output after uterotonic use in C-section under spinal anesthesia. They reported cardiac output increments up to 60% right after carbetocin or oxytocin administration, while in placebo patients, they only observed a 20% rise from basal values. Moreover, their parameters return almost to predelivery levels in 10 minutes [23].

During the 1970s, Niswonger et al. and Ueland et al. described higher cardiac output right after C-section and vaginal delivery and moderately returned to basal conditions after 60 minutes. Later studies in the 1980s and 1990s found the same cardiac output behavior: hyperdynamic at initial measurements during labor, marked increments during placenta delivery, and return to almost prelabor parameters 1 hour after delivery [25–29]. This clinical information may suggest that postpartum optimization is consequent to a reduction in heart workload rather than the concept of autotransfusion described in articles from the last century. This impression has been suggested recently by Kuhn et al. and Rosseland et al. However, other reports, such as Robson et al. [30], have still detected increased flow parameters 60 min and 24 h after delivery.

After the administration of epidural anesthesia, we also detected changes that were similar to those described by Dennis and Dyer, which included the decrease in systemic vascular resistance of up to 20%–40% from the initial values as early as during the first five minutes [31]. Other factors triggering SVRI alterations in our sample were carbetocin administration. These effects are long recognized, since the early 1990s, when researchers described the minimum effective dosages (8–30 *μ*g) [32]. They noted the consequent skin flushing and blood pressure alterations in nearly half the subjects evaluated. The clinical evidence placed carbetocin on specific scenarios for the management of the third postpartum period [33]. Recently the development of light and thermostable carbetocin formulations has engaged new interest, especially in the public sector [34], at doses of 80–100 *µ*g. However, Khan et al. had studied the pharmacological effects of these formulations and recommended even lower dosages (ED 90% 14.8 *μ*g), achieving acceptable uterine contraction and fewer side effects without increased blood loss [35].

Since 2014, our institution has implemented a goal-directed therapy approach as part of a perioperative strategy. The management integrates interventions for hemodynamic resuscitation with fluid volume or vasopressor drugs [36], central venous blood gas assessment of hematocrit and metabolic parameters [37], and viscoelastic evaluation to guide the transfusion of blood components [38–42]. The management strategy was based on studies akin to those published by Challand et al. in which benefits from goal-directed therapy were observed in high-risk surgical patients [43]. Furthermore, the adequacy of these goals, with the hemodynamic pattern as a guide, has been associated with shorter hospitalization times along with lower morbidity based on metanalyses [14, 44, 45].

From a hemodynamic point of view, our study also may suggest that active management preserves the cardiac behavior of pregnant patients who were under regional anesthesia through a procedure in which massive blood losses are anticipated. In this scenario, a highly skilled surgical team, proper nurse assistance, and administrative strategies with the blood bank are essential, and the anesthesia care team provides not only anesthesia management but also critical medical support to the patient.

## 6. Study Limitations

The present study had some limitations. There is a lack of data about patients who received general anesthesia for comparison with the time series in their respective parameters. Considering that general anesthesia for this surgery at our center is usually carried out in patients with preoperative obstetric hemorrhage, congenital or acquired coagulopathy, nonreassuring fetal status, a medical history that contraindicates a regional approach, or technique rejection, such conditions would render the parameters less comparable; the differences in the physical status between patients could bias the comparisons and return statistical results where the regional blockade holds less variance than the general technique.

Another limitation was the use of a single method for cardiac evaluation, which is calculated from the radial arterial waveform and the rest of the parameters derived from these approximations, without the resource of a pulmonary artery catheter for corroboration of the measurements from the minimally invasive monitor. Moreover, studies in obese patients with FloTrac indicate a moderate correlation between the derived cardiac output and results from the thermodilution technique through pulmonary artery catheterization and poor agreement during very high or low cardiac activity [46, 47].

## 7. Conclusions

In the present study, pregnant women in their third trimester underwent obstetric hysterectomy under epidural anesthesia. These patients had modest hemodynamic variance and less cardiac work requirements toward the end of the surgery. Also, we suggest that goal-directed therapy may preserve the hemodynamic stability despite the blood loss. Comparisons with patients in similar circumstances who had conserved their uterus or were under general anesthesia are needed to follow-up on this research.

## Figures and Tables

**Table 1:** Goal-directed therapy.

	Targets	Interventions
Hemodynamic		
Cardiac index^1^	At least 2.5 L/min/m^2^	Inotropes|Fluids
Systemic vascular resistance index^2^	1250–2500 dynes·sec·cm5/m^2^	Vasopressor|Fluids
Central venous pressure^3^	6–12 mmHg	Fluids
Mean arterial pressure^2^	65–90 mmHg	Vasopressor|Fluids

Thromboelastography		
R. Reaction time^4^	4–8 min	FFP 10 ml/kg
MA. Maximum amplitude^5^	62–84 mm	Cryoprecipitate 2 ml/Kg and platelet apheresis

Blood Test		
Hemoglobin^5^	70–90 gr/L	Packed red blood cells
Platelets^5^	<50 000 c/*µ*L	Platelet apheresis
Fibrinogen^6^	<200 mg/dL	Cryoprecipitate 2 ml/Kg
Prothrombin time^5^	<16	FFP 10 ml/kg
International normalized ratio^5^	<1.5	FFP 10 ml/kg

(1) C. Salzwedel, “Perioperative goal-directed hemodynamic therapy based on radial arterial pulse pressure variation and continuous cardiac index trending reduces postoperative complications after major abdominal surgery: A multicenter, prospective, randomized study,” Crit. Care, vol. 17, 2013. (2) J. G. Ouzounian, D. I. Masaki, T. K. Abboud, and J. S. Greenspoon, “Systemic vascular resistance index determined by thoracic electrical bioimpedance predicts the risk for maternal hypotension during regional anesthesia for cesarean delivery,” Am. J. Obstet. Gynecol., vol. 174, pp. 1019–1025, 1996. (3) A. Donati, “Goal-directed intraoperative therapy reduces morbidity and length of hospital stay in high-risk surgical patients,” Chest, vol. 132, pp. 1817–1824, Dec. 2007. (4) K. M. Antony, “Establishing thromboelastography with platelet-function analyzer reference ranges and other measures in healthy term pregnant women,” Am. J. Perinatol., vol. 32, pp. 545–553, 2015. (5) D. R. Spahn, “Management of bleeding and coagulopathy following major trauma: an updated European guideline,” Crit. Care, vol. 17, pp. 1–45, 2013. (6) A. J. Butwick and L. T. Goodnough, “Transfusion and coagulation management in major obstetric hemorrhage,” Curr. Opin. Anaesthesiol., vol. 28, pp. 275–284, 2015.

**Table 2:** Sample demographic characteristics of the participants.

		Mean, standard deviation
Age	Years	29.2 ± 3.4
Body Mass index	kg/m^2^	29.8 ± 2.8
Weeks of pregnancy	—	35.4 ± 1.7
Previous gestations	—	3.7 ± 1.7

*Operation room*		
Surgical procedure	Min	131.11 ± 26
Anesthesia	Min	198.8 ± 29
Ringer's lactate solution	mL	2966.67 ± 878.92
Saline 0.9%	mL	857.14 ± 377.96
Ephedrine boluses 5 mg	—	3.5 ± 2.8
Fresh frozen plasma	U	4.25 ± 0.46
Packed red blood cells	U	4 ± 1.22
Cryoprecipitate	U	10.8 ± 1.79
Platelet apheresis	U	5.1 ± 1.9*∗*
Blood loss	mL	3177.78 ± 733
Intensive care	Days	2.5 ± 1.2
Hospital stay	Days	3 ± 0.9

*∗*Bag platelet concentrate content.

**Table 3:** Hemodynamic parameters (mean, standard deviation).

Minutes	0	5	15	30	60	75	105	120	135	*P* value^*α*^
CO	7.8 ± 2.2	7.4 ± 2.7	7.9 ± 2.3	8.2 ± 2.2	7.8 ± 2	7.6 ± 2.1	6.9 ± 2.1	7.1 ± 2	7.2 ± 1.9	0.0494
CI	4.5 ± 1.3	4.3 ± 1.6	4.6 ± 1.3	4.8 ± 1.3^*β*^	4.5 ± 1.2	4.3 ± 1	4 ± 1.1	4 ± 1.1	4.1 ± 1.1	0.0571
SV	83.5 ± 24.6	79.8 ± 27.1	80.8 ± 23.5	84 ± 22.1	90.1 ± 27.6	93.9 ± 25.8^*β*^	86 ± 30.3	87.1 ± 31.5	83.2 ± 25.7	0.0062
SVI	47.7 ± 14.5	46.5 ± 15.8	46.8 ± 13.5	48.8 ± 13.1	51.6 ± 15.3	53.3 ± 13.8^*β*^	49.3 ± 17.7	49.6 ± 18.4	47.3 ± 15.2	0.015
HR	91.8 ± 15.9	93.7 ± 16	100.8 ± 16^*β*^	100.3 ± 18^*β*^	89.3 ± 18	83.6 ± 18	83.6 ± 17	85.2 ± 19	90 ± 21	0.0998
SVV	12.8 ± 10.8	10.8 ± 6	10.1 ± 4.8	9.5 ± 4.8	10.9 ± 5.6	11.4 ± 4.5	11.9 ± 5.3	11.8 ± 6.8	12.7 ± 7.2	0.6554
SVR	997 ± 344.2	1055 ± 393	887 ± 327	755 ± 250	844 ± 320	862 ± 266	920 ± 268	870 ± 239	927 ± 475	0.2593
SVRI	1973 ± 408	1786 ± 434	1517 ± 564	1288 ± 448	1471 ± 569	1502 ± 460	1592 ± 460	1524 ± 140	1621 ± 749	0.2501
MAP	92.6 ± 14.6	91.9 ± 15.5	90.9 ± 15.7	84.4 ± 13.7	87.2 ± 14	83.4 ± 11	82.8 ± 12.9	82.1 ± 14.3	85.6 ± 13.6	0.9583
CVP	11.6 ± 7.7	9.6 ± 7.7	10 ± 6.6	9.7 ± 4.9	10.6 ± 6.6	8.4 ± 5.6	7.5 ± 5.1	7.5 ± 4.7	8.1 ± 4.9	0.4886
CW	10.5 ± 3.5	9.8 ± 4.1	10.4 ± 3.5	10 ± 2.8	10 ± 3.5	9.2 ± 2.9	8.3 ± 2.7	8.6 ± 3.1	8.9 ± 2.8	0.0653
CWI	6 ± 1.9	5.7 ± 2.3	6 ± 2	5.8 ± 1.6	5.6 ± 1.7	5.2 ± 1.5	4.7 ± 1.5	4.9 ± 1.7	5 ± 1.6	0.0533
SW	111.6 ± 35.8	105.3 ± 39.1	107.6 ± 33.2	101.9 ± 28.3	114.9 ± 41	115.5 ± 38.7	105.1 ± 43.6	107.4 ± 47.2	104.2 ± 38.8	0.2363
SWI	63.9 ± 20.5	61.1 ± 22	62.1 ± 19.1	59.2 ± 16.2	65.4 ± 21.7	65.6 ± 21.1	60.6 ± 25.3	61.6 ± 27.5	59.3 ± 22.8	0.2116

Cardiac output, CO (L/min/m^2^); cardiac index, CI (L/min/m^2^); systolic volume, SV (mL), systolic volume index, SVI (mL/m^2^); heart rate, HR (b/min), stroke volume variation, SVV (%), systemic vascular resistance, (dynes·sec/·cm5); systemic vascular resistance index, SVRI (dynes·sec/cm5/m^2^); mean arterial pressure, MAP (mmHg); central venous pressure, CVP (mmHg); cardiac work, CW (kg × m); cardiac work index, CWI (kg × m/m^2^); stroke work, SW (gr × min); stroke work index, SWI (gr × min/m^2^). ^*α*^Repeated measures ANOVA. ^*β*^Post hoc <0.025.

## Data Availability

The data belongs to the Anesthesiology Department of Obstetrics and Gynecology Specialty Hospital No. 23, Mexican Social Security Institute, Monterrey, Nuevo León, Mexico. The information was requested via the Institution's ethics committee.

## Abbreviations

CI =: Cardiac index
CVP =: Central venous pressure
CWI =: Cardiac work index
*d* =: Cohen's d
MAP =: Mean arterial pressure
NS =: No significance (*P* > 0.05)
SVI =: Stroke volume index
SVRI =: Systemic vascular resistance index
SWI =: Stroke work index.

## Supplementary Materials

Supplementary Materials The supplementary material includes the analysis plan, additional tabular hemodynamic information, post hoc analysis *P* values, blood test data, hemodynamic values plotted, and their respective effect size calculations.
