# Supplementary material for: Minimally Invasive Hemodynamic Assessment during Obstetric Hysterectomy for Invasive Placentation with Epidural Anesthesia
Source: Anesthesiol Res Pract. 2020 Oct 28;2020:1968354. doi: 10.1155/2020/1968354 (PMC7641720; doi:10.1155/2020/1968354)
Supplement: Supplementary Materials — The supplementary material includes the analysis plan, additional tabular hemodynamic information, post hoc analysis P values, blood test data, hemodynamic values plotted, and their respective effect size calculations. [file 1968354.f1.docx]

**Supplementary Material**

| TABLE S1 | Hemodynamic Parameters (Post hoc P Values. 0 minutes as baseline) | | | | | | | | | | | | |
| --- | --- | --- | --- | --- | --- | --- | --- | --- | --- | --- | --- | --- | --- |
| Minutes | **0 min** | **5 min** | **15 min** | **30 min** | **45 min** | **60 min** | **75 min** | **90 min** | **105 min** | **120 min** | **135 min** | **F** | P |
|  |  |  |  |  |  |  |  |  |  |  |  |  |  |
| Cardiac Output | -- | 0.705 | 0.103 | 0.043 | 0.098 | 0.383 | 0.398 | 0.951 | 0.942 | 0.842 | 0.953 | 4.08 | 0.0494 |
| Cardiac Index | -- | 0.677 | 0.063 | **0.019** | 0.089 | 0.315 | 0.331 | 0.942 | 0.925 | 0.759 | 0.95 | 3.82 | 0.0571 |
| Stroke Volume | -- | 0.89 | 0.613 | 0.263 | 0.152 | 0.149 | **0.014** | 0.465 | 0.398 | 0.257 | 0.853 | 9.441 | 0.0062 |
| Stroke Volume Index | -- | 0.88 | 0.594 | 0.242 | 0.16 | 0.136 | **0.016** | 0.48 | 0.396 | 0.245 | 0.834 | 6.713 | 0.015 |
| S. Volume Variation | -- | 0.824 | 0.926 | 0.973 | 0.906 | 0.853 | 0.842 | 0.864 | 0.299 | 0.785 | 0.128 | 0.777 | 0.6554 |
|  |  |  |  |  |  |  |  |  |  |  |  |  |  |
| Systemic Vascular Resistance | -- | 0.457 | 0.688 | 0.958 | 0.722 | 0.778 | 0.723 | 0.252 | 0.064 | 0.657 | 0.087 | 1.4981 | 0.2593 |
| Systemic Vascular Resistance Index | -- | 0.543 | 0.32 | 0.043 | 0.289 | 0.233 | 0.289 | 0.751 | 0.939 | 0.336 | 0.917 | 1.5364 | 0.2501 |
| Heart Rate | -- | 0.715 | **0.009** | **0.022** | 0.573 | 0.911 | 0.999 | 0.9999 | 0.9999 | 0.993 | 0.846 | 2.94 | 0.09982 |
| Mean Arterial Pressure | -- | 0.654 | 0.807 | 0.993 | 0.987 | 0.985 | 0.998 | 0.994 | 0.998 | 0.999 | 0.996 | 0.28 | 0.9583 |
| Central Venous Pressure | -- | 0.923 | 0.804 | 0.824 | 0.772 | 0.979 | 0.986 | 0.954 | 0.976 | 0.978 | 0.954 | 0.7892 | 0.4886 |
|  |  |  |  |  |  |  |  |  |  |  |  |  |  |
| Cardiac Work | -- | 0.891 | 0.37 | 0.383 | 0.573 | 0.732 | 0.909 | 0.991 | 0.999 | 0.991 | 0.998 | 4.14 | 0.06531 |
| Cardiac Work Index | -- | 0.791 | 0.991 | 0.967 | 0.877 | 0.719 | 0.573 | 0.157 | 0.034 | 0.131 | 0.055 | 4.58 | 0.05337 |
| Stroke Work | -- | 0.949 | 0.828 | 0.748 | 0.556 | 0.442 | 0.248 | 0.809 | 0.849 | 0.748 | 0.971 | 1.964 | 0.2363 |
| Stroke Work Index | -- | 0.084 | 0.258 | 0.323 | 0.468 | 0.587 | 0.773 | 0.206 | 0.195 | 0.302 | 0.044 | 2.113 | 0.2116 |
|  |  |  |  |  |  |  |  |  |  |  |  |  |  |
| *Cardiac Output, CO (L/min/m²); Cardiac Index, CI(L/min/m²); Stroke Volume, SV (mL), Stroke Volume Index, SVI (mL/m²); Heart Rate, HR (b/min), stroke volume variation, SVV (%), Systemic Vascular Resistance, (dynes·sec·cm5); Systemic Vascular Resistance Index, SVRI (dynes·sec·cm5/m²); Mean Arterial Pressure, MAP (mmHg); Central Venous Pressure, CVP (mmHg); Cardiac Work, CW (kg x m); Cardiac Work Index, CWI (kg x m/m²).*  *Stroke Work, SW (gr x min); Stroke Work Index, SWI (gr x min/m²).*  *α Repeated Measures ANOVA.*  *F Repeated Measures ANOVA statistic.* | | | | | | | | | | | | | |

|  |  |  |  | |  |  |  |  |
| --- | --- | --- | --- | --- | --- | --- | --- | --- |
|  | **Table S2** |  |  | |  |  |  |  |
|  | **Blood Tests** |  | **Preoperative values** | **Postoperative values** |  | P value^α^ |  |  |
|  |  |  | **Mean, Standard Deviation** | **Mean, Standard Deviation** |  |  |  |  |
|  |  |  |  |  |  |  |  |  |
|  | Hemoglobin level | g/L | 106 ± 7 | 105 ± 8 |  | 0.906 |  |  |
|  | Hematocrit level | % | 34.4 ± 1.6 | 32.1 ± 3 |  | 0.025 |  |  |
|  | Platelet count | 10^9^/L | 224 ± 40.1 | 163 ± 32.9 |  | 0.008 |  |  |
|  | Prothrombin Time | s | 13.4 ± 0.8 | 14.4 ± 1.2 |  | 0.017 |  |  |
|  | Thromboplastin Time | s | 26.4 ± 5.3 | 30.4 ± 12.2 |  | 0.859 |  |  |
|  | Fibrinogen level | mg/dL | 507.3 ± 228.9 | 519.1 ± 91.7 |  | 0.753 |  |  |
|  | International Normalized Ratio |  | 1.1 ± 0.19 | 1.13 ± 0.1 |  | 0.514 |  |  |
|  | Glucose level | mg/dL | 86.1 ± 20.5 | 105.6 ± 20.5 |  | 0.018 |  |  |
|  | Creatinine level | mg/dL | 0.49 ± 0.09 | 0.46 ± 0.1 |  | 0.753 |  |  |
|  | BUN level | mg/dL | 8 ± 2.31 | 7.4 ± 1.8 |  | 0.109 |  |  |
|  | Urea level | mg/dL | 16.7 ± 4.6 | 14.1 ± 5.4 |  | 0.249 |  |  |
|  |  |  |  |  |  |  |  |  |
|  | ^α^*t-test* |  |  |  |  |  |  |  |

| 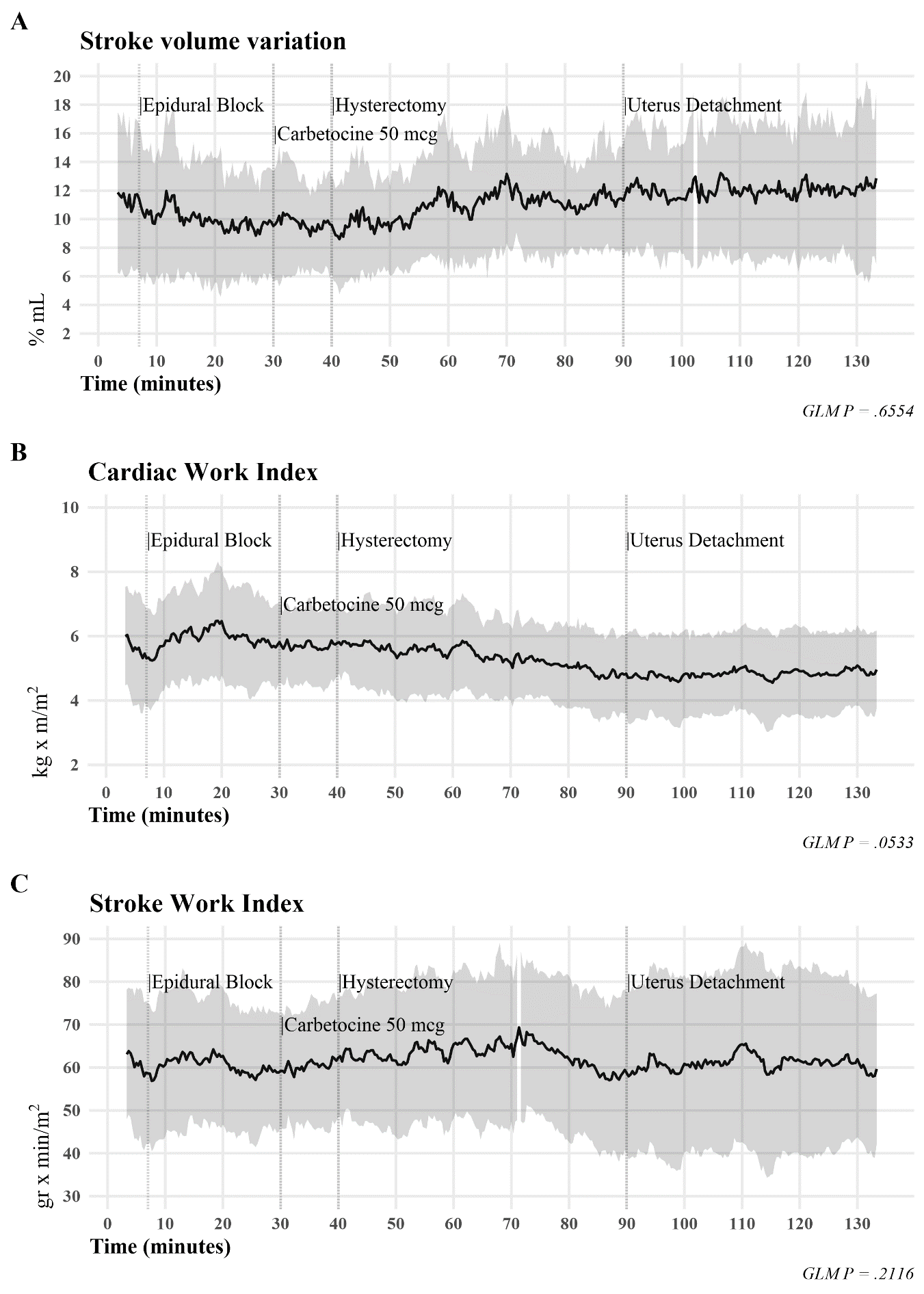 |
| --- |
| **Figure S1. Hemodynamic trends. (**x̄, bold black line; σ, gray zone) Stroke volume variation **(A)** showed minor variance, also, was not used as a parameter for fluid or vasopressor intervention during the management of the patients. The cardiac work index **(B)** displayed a slow decline as the uterine and systemic circulation were separated. However, the stroke work **(C)** index remained almost at the same values during the surgery. |

| 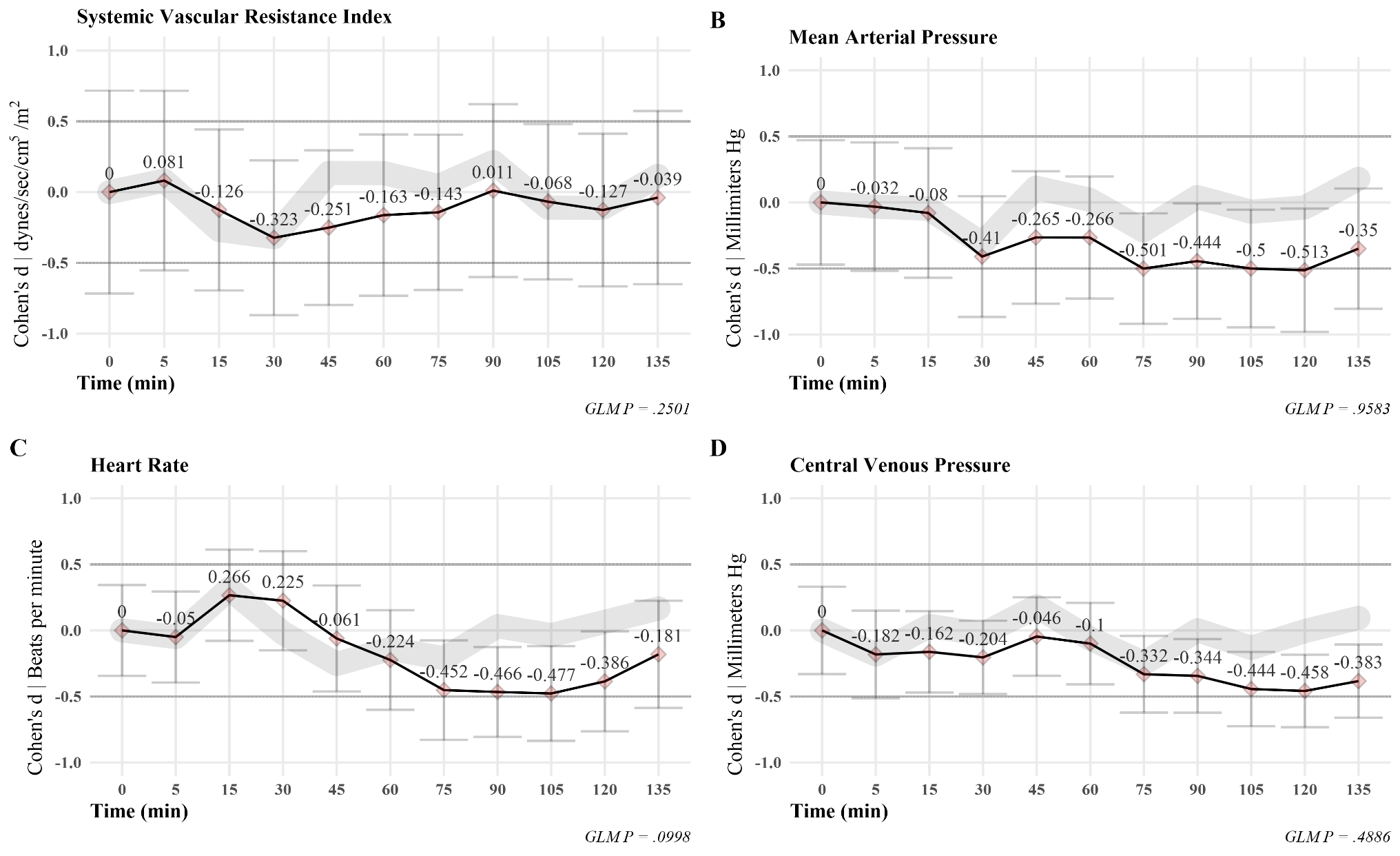 |
| --- |
| **Figure S2. Effect size (Cohen's d) assessment**. The bold black line represents the changes (Cohen's d) with respect to the initial measurements; the differences between the time intervals are represented in gray color. Systemic vascular resistances **(A)** had relatively mild (*d = -0.3*) differences after the epidural anesthesia and displayed lower variance during the rest of the surgery, despite the blood loss. Coincidental with the vascular resistances behavior, the mean arterial pressure **(B)** also declined after the epidural anesthesia, nonetheless continued below the baseline values during the procedure at the same size of change with no statistical significance. Heart Rate **(C)** increased right after the epidural anesthesia to meet the flow demands with a high cardiac output. After the initial hemodynamic adjust, its behavior changed to a lower measurement than its initial values during the hysterectomy stage. Central venous pressure **(D)** showed limited variation. |

**Acknowledgments Section**

**Research Staff**

Alvarado Ramos, Salomón

Study design, planning, statistical analyses, and study report.

Lara Díaz, Víctor Javier

Methodological and statistical counseling.

López Gutiérrez, María del Rosario

Medical expertise, management, and monitor study performance.

Torcida González, Miguel Eloy

Medical expertise, quality assurance, and quality control.

Campos Rodríguez, Jorge Francisco

Study design and protocol compliance with the review board committee.

**Departments**

UMAE 23 Department of Anesthesiology, Gynecology, Neonatology, Intensive Care and Nursing
